# Nano-indentation reveals a potential role for gradients of cell wall stiffness in directional movement of the resurrection plant *Selaginella lepidophylla*

**DOI:** 10.1038/s41598-019-57365-z

**Published:** 2020-01-16

**Authors:** Meisam Asgari, Véronique Brulé, Tamara L. Western, Damiano Pasini

**Affiliations:** 1 Department of Mechanical Engineering, McGill University, 817 Sherbrooke Street West, Montréal, QC H3A 0C3 Canada; 2 Theoretical and Applied Mechanics Program, School of Engineering and Applied Science, Northwestern University, 2145 Sheridan Rd., Evanston, IL 60208-3109 USA; 3 Department of Biology, McGill University, 1205 Avenue Docteur Penfield, Montréal, QC H3A 1B1 Canada

**Keywords:** Cell wall, Mechanical engineering

## Abstract

As a physical response to water loss during drought, inner *Selaginella lepidophylla* stems curl into a spiral shape to prevent photoirradiation damage to their photosynthetic surfaces. Curling is reversible and involves hierarchical deformation, making *S*. *lepidophylla* an attractive model with which to study water-responsive actuation. Investigation at the organ and tissue level has led to the understanding that the direction and extent of stem curling can be partially attributed to stiffness gradients between adaxial and abaxial stem sides at the nanoscale. Here, we examine cell wall elasticity to understand how it contributes to the overall stem curling. We compare the measured elastic moduli along the stem length and between adaxial and abaxial stem sides using atomic force microscopy nano-indentation testing. We show that changes in cortex secondary cell wall development lead to cell wall stiffness gradients from stem tip to base, and also between adaxial and abaxial stem sides. Changes in cortical cell wall morphology and secondary cell wall composition are suggested to contribute to the observed stiffness gradients.

## Introduction

Nature is a perpetual source of inspiration for biomimetic and actuating devices^1^. Current biomimetic research involves a multi-scale approach to investigate how structural and mechanical properties at various length-scales determine organism function^2^. As more hierarchical models are studied, and their micro- and nanoscale properties better understood, more complex biomimetic and actuating devices can be designed^2,3^. Of the many organisms studied, plants are particularly interesting because of the wide range of functions and structures produced from a limited set of constituent materials, i.e. cell wall components^4,5^. Juxtapositions of different tissues and/or cell wall structures and compositions can drive organ movements in response to water or humidity. These include both rapid, ‘active’ actuation in response to changes in cellular turgor pressure in living tissues, or slower, ‘passive’, hygroscopic movements resulting from the shrinking and swelling of the cell walls themselves in living or dead organs^6–8^.

**Figure 1:**
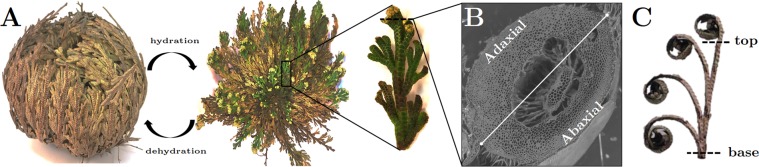
Water-Responsive Deformation in *S*. *lepidophylla*. (**A**) Mature *S*. *lepidophylla* plant showing dehydrated and hydrated conformations, as well as an isolated inner stem (inset). When dehydrated, *S*. *lepidophylla* appears as a ball of outer stems curled over its inner stems. When hydrated, stems are uncurled and lie flat in a spiral rosette; (**B**) Transverse section from the apical region of an inner stem imaged with scanning electron microscopy. Adaxial (upper) and abaxial (lower) stem sides are indicated, with a white line bisecting the transition area. (**C**) Time-lapse images showing the pattern of reversible inner stem curling. As stems dry, the tip curls on itself toward the stem base to form a spiral conformation. Upon rehydration with water, this process is reversed.

*Selaginella lepidophylla*, a desiccation tolerant spikemoss, is a promising hierarchical model to study hygroscopic actuation^9–11^. *S*. *lepidophylla* plants consist of hundreds of stems developing in a spiral phyllotaxy that curl upon themselves when dehydrated (Fig. 1A,C). Curling is a physical mechanism to prevent thermal and photoirradiation damage to photosynthetic surfaces during prolonged periods of drought^12,13^. During desiccation, the stem tip curls toward the upper (adaxial) side, and away from the under (abaxial) side (Fig. 1B,C). Stem curling/uncurling is driven by differential swelling and shrinking of adaxial and abaxial stem sides in response to water gain or loss^10,11^. The main tissue involved in this process is the cortex, which comprises the bulk of the *S*. *lepidophylla* stem (Fig. 1B)^11^. A thin epidermal layer and an amphicribral vascular bundle are also present; however, these tissues do not significantly contribute to the differential swelling and shrinking that drives stem curling/uncurling. Stiffness, defined as the ability of a material to resist deformation in response to an applied stress, determines the direction and extent of conformational changes undertaken by a material and hence is a critical mechanical property governing actuation response. The stiffer a material is, the less it deforms under a mechanical load. By layering or otherwise locally joining materials with dissimilar stiffness in a composite material, it is possible to control the global deformation in a specific direction^14,15^. Stiffness profiles leading to deformation in *S*. *lepidophylla* have been studied at the organ and tissue level^10,11^. Here, we present a complementary nanoscale investigation that sheds light on previously unexplored factors controlling the stiffness of *S*. *lepidophylla* in static and time-varying loads. The specific focus is on the elastic moduli of cortical cell walls, and the aim is to understand their contribution to the observed direction and degree of curling of inner stems. We take advantage of atomic force microscopy (AFM), a technique frequently used to study plant cell wall mechanics^16–18^. In particular, we use AFM indentation to locally measure the nanoscale elastic properties of cell walls, as well as to assess the level of inhomogeneity and stiffness gradients of the tissue across representative transverse sections of the plant, both longitudinally (stem tip to base) and between adaxial and abaxial stem sides. We also preliminarily investigate the viscoelastic behaviour at given loading rates.

## Materials and Methods

### Plant materials

Mature *S*. *lepidophylla* plants were purchased from Canadian Air Plants (New Brunswick, Canada), and maintained in a desiccated state at 25 °C and ~30–50% relative humidity until use. Prior to experimentation, *S*. *lepidophylla* plants were placed in a plate of water and allowed to rehydrate for three consecutive days to achieve 100% relative water content.

### Time-lapse video capture

Video capture was adapted from the protocol in Rafsanjani *et al*.^10^. Time-lapse videos were captured using a Logitech C920 HD Pro Webcam (1080p, Carl Zeiss optics) and Video Velocity Time-Lapse Studio software (Candylabs). *S*. *lepidophylla* plants were allowed to rehydrate for 24 hours. Individual inner stems were cut from the plant at the root-stem interface and secured in a metal clamp, which was affixed to the base of a square Petri dish. Stems were then allowed to air-dry for approximately 6 hours, during which time changes in their curvature were captured via time-lapse filming at frame rate of 1 min 21 s. Stems were taken from four different *S*. *lepidophylla* plants. In total, four inner stems were tested and displayed similar curling/uncurling patterns.

### Histochemical and immunological analysis of cell wall composition

A total of ten inner stems from five individual plants were isolated and cut into five regions (top, top-middle, middle, middle-base, and base). For lignin detection and cell wall counter-staining, samples were fixed with FAA (formaldehyde:acetic acid:alcohol) solution for one week, dehydrated through an ethanol series, and embedded in paraffin. Following paraffin removal, thick sections (1 μm) were stained with safranin O and alcian blue as outlined in Ruzin^19^.

A second set of ten inner stems from five individual plants were isolated and cut into five regions for immunohistochemical investigation. For pectin and hemicellulose detection, samples were fixed and embedded in London Resin White following the method outlined in Young *et al*.^20^. Samples were incubated at room temperature in a blocking solution [5% (w/v) normal goat serum (NGS) in 1× Tris-buffered saline/0.2% Tween (v/v) (TBST)] in a homemade humidity box for 40 minutes. Blocking solution was washed off using 1× TBST. Slides were then incubated with primary antibodies at 1:10 dilution (v/v) in 1% (w/v) NGS blocking solution in the humidity box for 1 hour (antibodies are described below). Slides were washed 2 × 20 minutes in 1× TBST. Secondary antibodies were diluted at 1:100 (v/v) in 1% (w/v) NGS blocking solution in the dark for 45 minutes. Slides were washed 2 × 20 minutes in the dark and mounted in 90% (v/v) glycerol. Control slides were used to test the specificity of the secondary antibodies and also to test for autofluorescence. Blocked slides that were not incubated with primary or secondary antibodies were imaged, as well as slides blocked and incubated with only secondary antibody. LM10 (Rat IgG2c) and LM11 (Rat IgM) antibodies were obtained from Plant Probes, UK^21^. LM10 binds to unsubstituted or low-substituted xylan backbone chains, while LM11 is able to additionally bind to wheat arabinoxylan. JIM7 (Rat IgA) and JIM13 (Rat IgM) were obtained from the Complex Carbohydrate Research Centre^22,23^. JIM7 binds to partially methyl-esterified homogalacturonan and JIM13 binds to arabinogalactan and arabinogalactan protein. Alexa-fluor 488 secondary antibody was obtained from Invitrogen (goat anti-Rat IgG (H + L) polyclonal, CAT# A-11006).

For cellulose detection, a total of five, fully hydrated *S*. *lepidophylla* stems were isolated from three different plants and embedded in polyethylene glycol (PEG) using the protocol from Gierlinger *et al*.^24^. Embedded samples were sectioned (10 μm thickness) using a Leica RM2245 semi-automated rotary microtome. Solidified PEG was then removed using washes of ddH_2_O. Samples were then prepared following the method outlined in Ruzin^19^ for calcofluor white staining.

All samples were imaged using a Leica DM6000B epifluorescence microscope (Leica Microsystems, Wetzlar Germany) and QImaging Retiga CCD camera with Openlab imaging software (QImaging, British Columbia Canada).

### Scanning electron microscopy

Fresh, hand-cut sections (~1 mm thick) from the apical stem region were mounted on SEM stubs and allowed to air-dry (at a relative humidity ~30%) for 24 hours prior to imaging. Dry sections were imaged using a Hitachi TM3030Plus SEM in backscatter electron mode.

### Atomic force microscopy

A JPK Atomic Force Microscope (JPK Nano-wizard@3 Bio Science, Berlin, Germany) was used for imaging and force spectroscopy. *S*. *lepidophylla* stem samples (three stems, each from separate plants, with five regions per stem, and three replicate sections per region) were cut transversally using a sharp blade in a wet state. Sections (1 mm in thickness) were air-dried to ambient humidity (~30% relative humidity [RH]) and placed on double-sided clear tape on a microscope slide. Cortical stem tissue in adaxial and abaxial regions was located to perform force measurements. All the measurements were performed on tissue in ~30% RH. Using the QI imaging mode of the JPK AFM, a number of force maps were created within the area of 30 μm × 30 μm on each sample. Reduced scan areas were then selected to obtain the structural details of the cell walls in which 128 × 128 indentation points were tested in areas of 1–10 μm^2^. The force maps averaged 3 μm^2^ in size. For consistency we prescribed the indentation parameters (e.g. window size, number of points, indentation depth) within the cortical tissue area across stem sections and between adaxial and abaxial sides.

Three types of AFM probes were used for contact mode imaging: (1) Non-conductive silicon nitride cantilevers with integrated conical tips of radius 20 nm (MLCT Micro-cantilever, Bruker, Mannheim, Germany) [a spring constant of 0.06 N/m and a resonance frequency of 22 kHz]; (2) Biotool high resolution qp-BioAC/Quartz cantilevers with a 2 nm defined conical tip (Nanotools USA LLC, Henderson, NV) [a 60 μm length, a spring constant of 0.1 N/m and a nominal resonance frequency of 50 kHz]; and (3) Super-sharp standard Force Modulation Mode Reflex Coating (FMR) cantilevers with diamond-like carbon nano-tip of radius 2–3 nm (Nanotools USA LLC, Henderson, NV) [a spring constant of 2.8 N/m, a nominal resonance frequency of 75 kHz in air]. Non-Contact High Resonance (NCHR) cantilevers (Nanotools USA LLC, Henderson, NV) with a nominal spring constant of 40 N/m and integrated spherical tip of radius 100 nm (±10%) were applied for indentation measurements. The indentation frequency was 1–500 Hz. The AFM measurements were performed in an ambient environment of 20–25 °C and ~30% RH. Prior to each indentation test, the deflection sensitivity of the AFM cantilever was calibrated by engaging the cantilever on the surface of a clean microscope slide^25^. The spring constant of the cantilever was then determined from the power spectral density of the thermal noise fluctuations in air by fitting the first free resonance peak of the AFM cantilever to that of a simple harmonic oscillator using the JPK software^26^.

Indentations were repeated at given locations to ensure that no permanent deformation occurred at the surface of the sample. Force maps containing 128 × 128 indentation points were created on each indentation area. The elastic modulus *E* of a sample was obtained from the retracting force-indentation depth curve through Hertzian contact mechanics, where $E=3F(1-{\nu }^{2})/4\sqrt{R{\delta }^{3}}$ is the relation between the elastic modulus *E* and the applied indenting load *F* with $\nu$ being the Poisson’s ratio of the sample, *R* the radius of the AFM probe, and *δ* the indentation depth^27^. A number of assumptions were considered. The deformation of the sample relative to its thickness and also relative to the radius of the probe was assumed very small^28^. Any strain below the elastic limit was also assumed infinitesimal, a condition satisfied with the use of an indentation depth below 50 nm that rules out the influence of the glass substrate as well as any nonlinear and inelastic behaviour of the tissue at higher strains^29,30^. The Poisson’s ratio $\nu$ was selected to be 0.5. Data analysis was performed with the JPK data processing software. Statistical significance was determined by a paired Student’s t-test, when applicable. Differences were considered significant at p < 0.05.

## Results and Discussion

### Cell wall appearance differs between adaxial and abaxial stem sides

**Figure 2:**
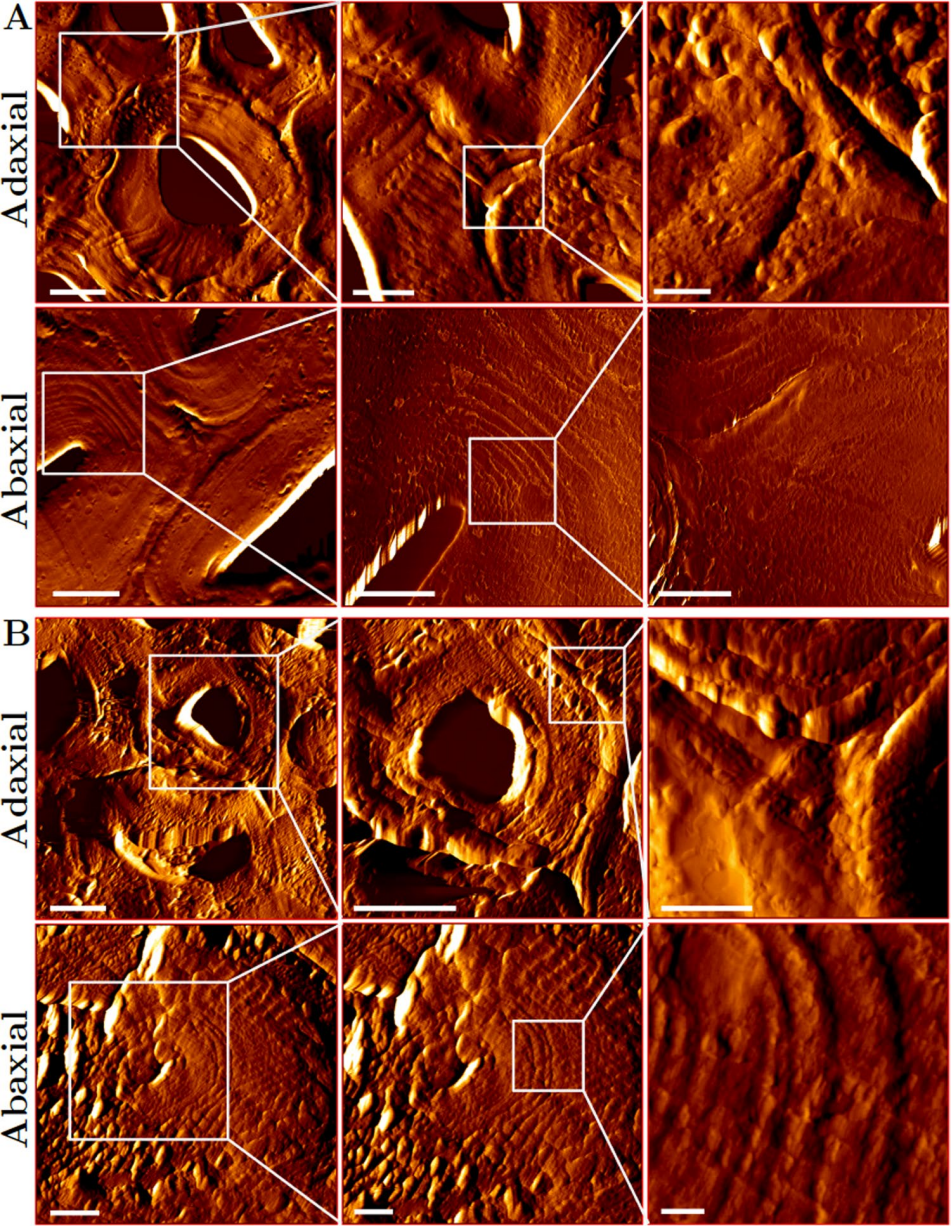
Cell Wall Layering in *S*. *lepidophylla* Cortex. AFM topological scans of apical (**A**) and basal (**B**) regions showing adaxial and abaxial cell shape and cell wall layering. Abaxial cell walls in both apical and basal stem regions show more prominent layering than in adaxial cell walls. Scale bars from left to right represent 5 μm, 2 μm, and 500 nm.

Dried, transverse sections from a set of regions along the length of inner *S*. *lepidophylla* stems were scanned with AFM to generate topographical images of the adaxial and abaxial cortical cell walls (Fig. 2A,B; Supplementary Fig. 1). Overall, cortex cells appear round to oval in geometry, with no obvious change in cell shape between adaxial and abaxial stem sides or between tip and basal stem segments. Differences arise when comparing cell wall layering between adaxial and abaxial cortical cells. Abaxial cell walls show very distinct secondary cell wall layers, while those in the adaxial region appear relatively smooth. This pattern is observable in sections along the length of the stem and is also visible in cortical cell walls imaged with transmission electron microscopy (Supplementary Fig. 2). Typical dicot and gymnosperm secondary cell walls contain three layers (S1–S3, with S2 being the thickest layer)^31–33^. In some monocots, such as bamboo species, more layers are visible. Bamboo fibers can have up to six to eight distinct cell wall layers^33–36^. Qualitatively, *S*. *lepidophylla* cortical cells walls, especially those on the abaxial stem side, appear to have more than three SCW layers, and therefore resemble bamboo with respect to cell wall layer morphology. Compared to adjacent cell types, fiber cells in *Arabidopsis thaliana*, wood, and bamboo are stiffer and mechanically support the stem/trunk/culm^37–40^. Thus, given their secondary cell wall morphology, *S*. *lepidophylla* stem cortical cells most likely act like fiber cells, providing structural support and reinforcement to the stem.

### Cell wall elasticity differs between adaxial and abaxial stem sides

**Figure 3:**
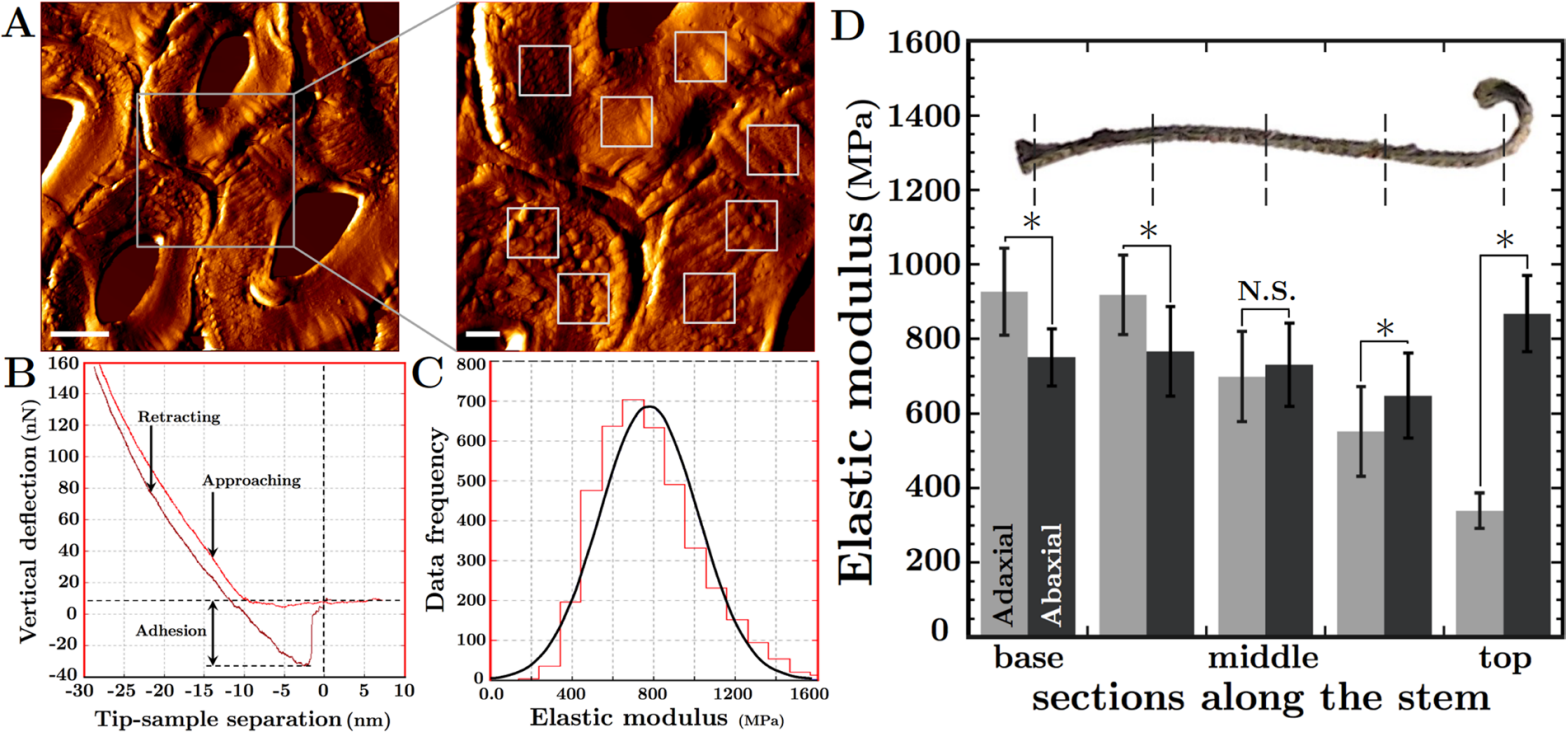
Stiffness of Cortical Cell Walls in the *S*. *lepidophylla* Stem. (**A**–**C**) Representative AFM results from scanning and indenting an apical cortical cell from an inner stem at the indentation frequency of 250 Hz. (**A**) Topological scan showing the cell walls of three neighbouring adaxial cells and the areas chosen for indentation (inset). Eight representative force maps within an apical adaxial cortical cell from an inner stem are shown. 128 × 128 indentation points were tested in areas of 1–10 μm^2^. Scale bars from left to right represent 5 μm and 2 μm. (**B**) A typical force spectroscopy curve for a single indentation point showing adhesion forces of ~40 nN (distance of detachment ~30 nm). Both approaching and retracting curves are shown; (**C**) Histogram showing the average distribution of elastic modulus *E* across cell wall indentations in a single area. The red graph represents the experimental measures, and the solid dark curve is the Gaussian curve of the best fit; (**D**) Image showing the five regions along the length of inner stems in a dry state used for AFM indentation analysis and a graph of the results obtained in each region for the adaxial versus abaxial sides of the stem. Significant differences between stem sides within each point are indicated by $\ast$ (p < 0.05; Table 1). N.S. represents no significant difference (p > 0.05).

Cross-sections from five regions were selected, and indentation responses of cortical cell walls were retrieved at 250 Hz for both adaxial and abaxial areas, thus offering a means of comparison between the two stem sides (Fig. 3; Table 1). Five to ten cells were tested in each region and stem side. Between 4096 to 16384 points were examined for each cell wall (Fig. 3A). Graphs of indentation force and elastic modulus distribution were generated for each indented position in a given cell wall. Figure 3B illustrates the interaction between the AFM probe and the tissue as the probe is pushed on the sample surface up to a certain indentation depth; the area within their loading and unloading paths denotes the dissipation energy. In the apical stem region, for a given indentation depth on any section, the maximum average force measured within an abaxial cortical cell (~200–250 nN) was above the value obtained for an adaxial cortical cell (~100–140 nN), indicating higher stiffness of abaxial tissue relative to adaxial tissue. The distributions on these graphs were consolidated and an average longitudinal elastic modulus *E* for adaxial and for abaxial cortical cell walls for each stem region was calculated (Fig. 3B–D; Table 1). Significant stiffness differences were observed between adaxial and abaxial cell walls along the length of the stem, with the exception of the middle stem sections in which *E* was comparable between adaxial and abaxial regions. Interestingly, while the value of *E* in abaxial cortical cell walls remained roughly consistent along the length of the stem (~700–800 MPa), it was found to decrease steadily in adaxial cell walls moving from the mid-base region (~920 MPa) to the tip of the stem (~340 MPa) (Fig. 3; Table 1).

**Table 1:** *S*. *lepidophylla* Cortical Cell Wall Elasticity.

Cortical Tissue	Elastic Modulus Top (MPa)	Elastic Modulus Top-Middle (MPa)	Elastic Modulus Middle (MPa)	Elastic Modulus Middle-Base (MPa)	Elastic Modulus Base (MPa)
Adaxial	339.28 ± 13.29* p = 0.00005	550.74 ± 15.19* p = 0.00009	699.38 ± 21.81	919.00 ± 14.63* p = 0.00136	927.08 ± 21.12* p = 0.00144
Abaxial	867.52 ± 31.50* p = 0.00005	633.72 ± 19.96* p = 0.00009	718.96 ± 27.71	778.61 ± 14.27* p = 0.00136	738.34 ± 23.84* p = 0.00144

Five adaxial and five abaxial cells were indented for each stem region (apical [top], top-middle, middle, middle-basal and basal) at 250 Hz. Differences between adaxial and abaxial stem regions were tested using two-sided Wilcoxon sign-rank tests with a cut-off of p = 0.05 and significant results are marked by *. Data are shown in terms of mean ± standard error. At the stem base, adaxial cortical cell walls are significantly stiffer (1.44 e-03, p < 0.05) than abaxial cell walls, whereas the opposite is observed at the stem tip (5.02 e-05, p < 0.05).

The gradient of stiffness in adaxial cortical cell walls follows a secondary cell wall developmental (SCW) gradient in which walls become thicker and increasingly lignified moving from stem tip to base, as reported in monocots such as bamboo, and eudicots such as *A*. *thaliana* and various tree species^37,38,41^. This type of stiffness gradient allows the upper portions of stems/trunks/culms to be flexible and bend more readily in response to environmental factors such as wind, while the stiffer base resists movement and prevents the plant from being uprooted^42–44^. What is novel about *S*. *lepidophylla* is that there is also an adaxial to abaxial gradient of SCW stiffness that likely promotes directional bending toward the adaxial side. These SCW stiffness changes are paired with differences in cell morphology and orientation, suggesting alternate development between the two sides of the stem^11^. This could be akin to the differential regulation of adaxial and abaxial developmental programs essential for correct patterning of planar organs such as leaves in other plant species, such as *A*. *thaliana*^45^. Based on such a model, differences in the onset and extent of SCW differentiation could lead to a steeper gradient of stiffness from base to tip in adaxial stem cortex, and a relatively subtler gradient in abaxial stem cortex. Taken together, in *S*. *lepidophylla*, these two perpendicular SCW stiffness gradients (longitudinal and adaxial-abaxial) are expected to work together to allow the stem tip to curl tightly on itself toward the adaxial side (least stiff region of the stem) while restricting movement at the stem base, and to direct movement away from the abaxial side (Fig. 1C)^10,11^.

A number of considerations need to be made to interpret the results obtained from AFM and other indentation techniques^17,46,47^. Of particular importance is the indentation depth and force relative to the thickness of the specimen. If the indentation is too deep or the forces are too large, there is a risk that the sample could be significantly damaged and/or that the observed results were influenced by the properties of the substrate underlying the section (i.e., a glass slide). To minimize these factors, the samples were indented with low magnitude forces, below 500 nN, and the indentation depth chosen (~20–30 nm) was smaller than both the thickness of the sample (~1 mm) and the radius of the indenting sphere (~100 nm). Another factor that must be considered when indenting living plant cell walls is the contribution of turgor (internal cell pressure on the cell wall from the fluid-filled vacuole) to the measured mechanical properties^47–49^. Inner *S*. *lepidophylla* stems, still undergoing development, are living^9,11^. Thus, to reduce the influence of turgor on the measured cell wall *E* of cortical cells, samples were air-dried to a relative water content of ~5% prior to indentation.

### Cortical cell walls are viscoelastic and show increasing stiffness at higher frequencies

**Figure 4:**
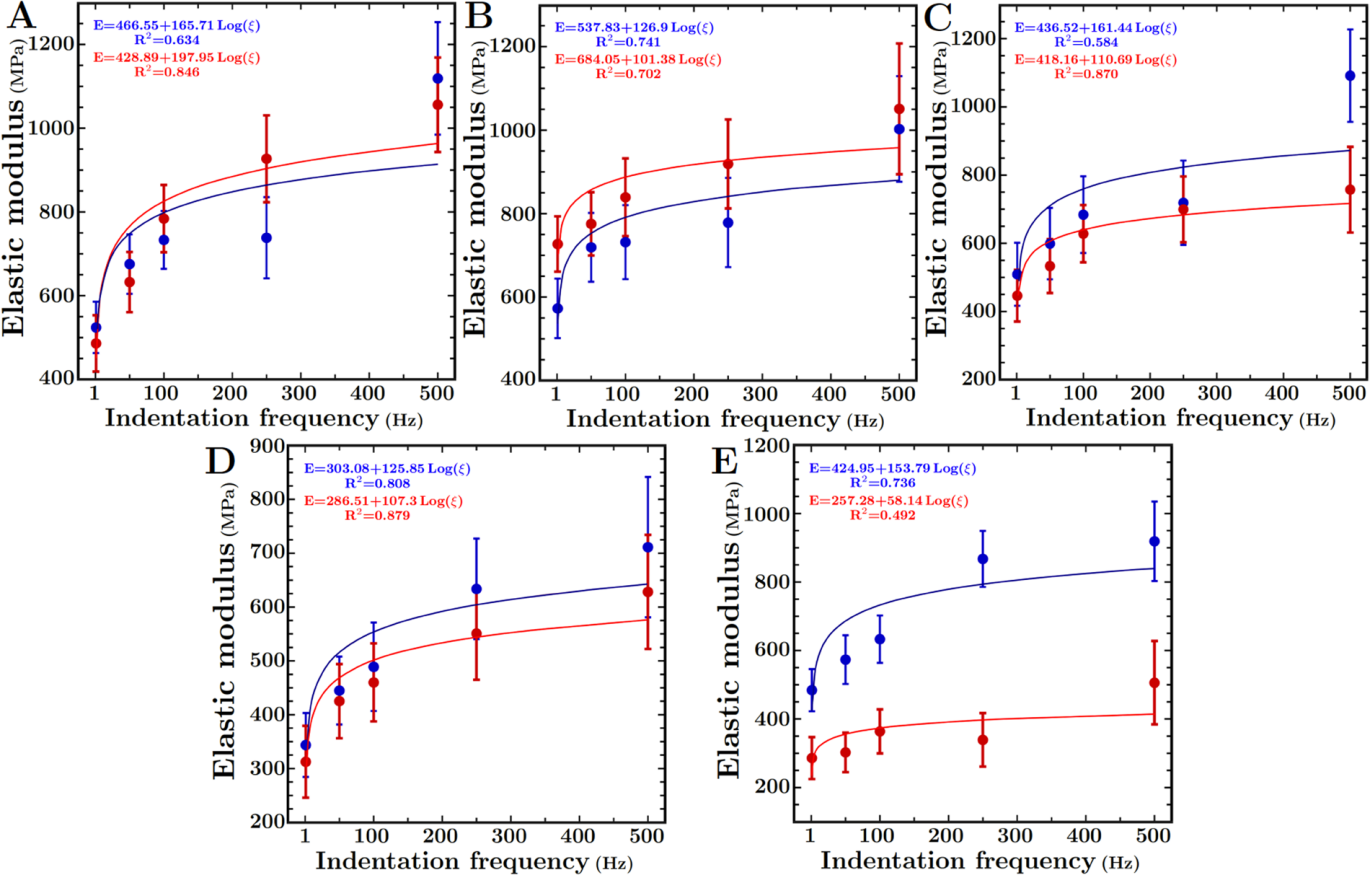
Elastic Modulus of Stem Regions Against Indentation Frequency. (**A**–**E**) Measured elastic moduli of adaxial and abaxial areas of basal (**A**), middle–basal (**B**), middle (**C**), apical–middle (**D**) and apical (**E**), regions at indentation frequencies of 1–500 Hz (Mean ± SD). The plots show the mean and standard error of the measured elastic modulus *E* of all samples for given indentation rate 1/$({T}_{e}+{T}_{r})$, where ${T}_{e}$ is the extending time and ${T}_{r}$ the retracting time of the probe during indentation, which were set equal at each loading rate^28,29^. Adaxial elastic moduli in MPa are plotted with a logarithmic fit in red, while abaxial results are shown in blue. For apical stem, the mean value of the abaxial elastic modulus increased from ~500 MPa at 1 Hz to ~850 MPa at 500 Hz, showing more than ~70% increase. The mean value of the adaxial elastic modulus in the same region increased from ~300 MPa at 1 Hz to ~500 MPa at 500 Hz, showing more than ~65% increase. For the basal part of the stem, the increase in abaxial elastic modulus was more than 100% from ~500 MPa at 1 Hz, to ~1100 MPa at 500 Hz. In the same region, the increase in adaxial elastic modulus was about 100% from ~480 MPa at 1 Hz, to ~1050 MPa at 500 Hz.

Since biological materials are complex and often exhibit viscoelastic behaviour, we investigated the loading rate dependency of the elastic behaviour of *S*. *lepidophylla* cell walls. Additional indentation tests were performed at five indentation frequencies (1, 50, 100, 250 and 500 Hz) on the regions and areas previously tested (Fig. 4)^28,50^. Similar to the testing at a single frequency [250 Hz], the elastic moduli of abaxial (lower) cell walls remained relatively constant along the length of the stem, while those of the adaxial cortical cell walls were significantly lower in the apical region compared to the stem base. For a given indentation depth, *δ*, below 50 nm, *E* increased with the indentation frequency, showing the dependence of tissue stiffness on loading rate. Further, for each prescribed indentation depth, the maximum indentation load increased with the loading frequency, confirming high values of the elastic modulus at high loading rates. The change in elastic modulus at given indentation frequencies is typical of biological samples and suggests that *S*. *lepidophylla* cortical cell walls along the length of the stem are viscoelastic^28,29,50^. The large standard errors of the experimental data, in particular at high frequencies, can be attributed to tissue heterogeneity, which varies with position in all regions within the stem. Our results are consistent with the viscoelastic behaviour observed with nano-indentation, including AFM, of dried secondary cell walls in flax fibres and wood^51–53^.

### Cell wall composition resembles gelatinous fibers of tension wood, coiling vines, and ice plant seed capsules

It is well established that cell wall composition affects cell wall mechanical properties, including stiffness^31,54,55^. We thus examined cortical cell wall composition along the length of inner *S*. *lepidophylla* stems on both adaxial and abaxial sides. We were particularly interested in identifying the existence of any relation between the cell wall composition and the cell wall layering patterns observed with AFM (Fig. 3) and TEM (Supplementary Fig. 2).

**Figure 5:**
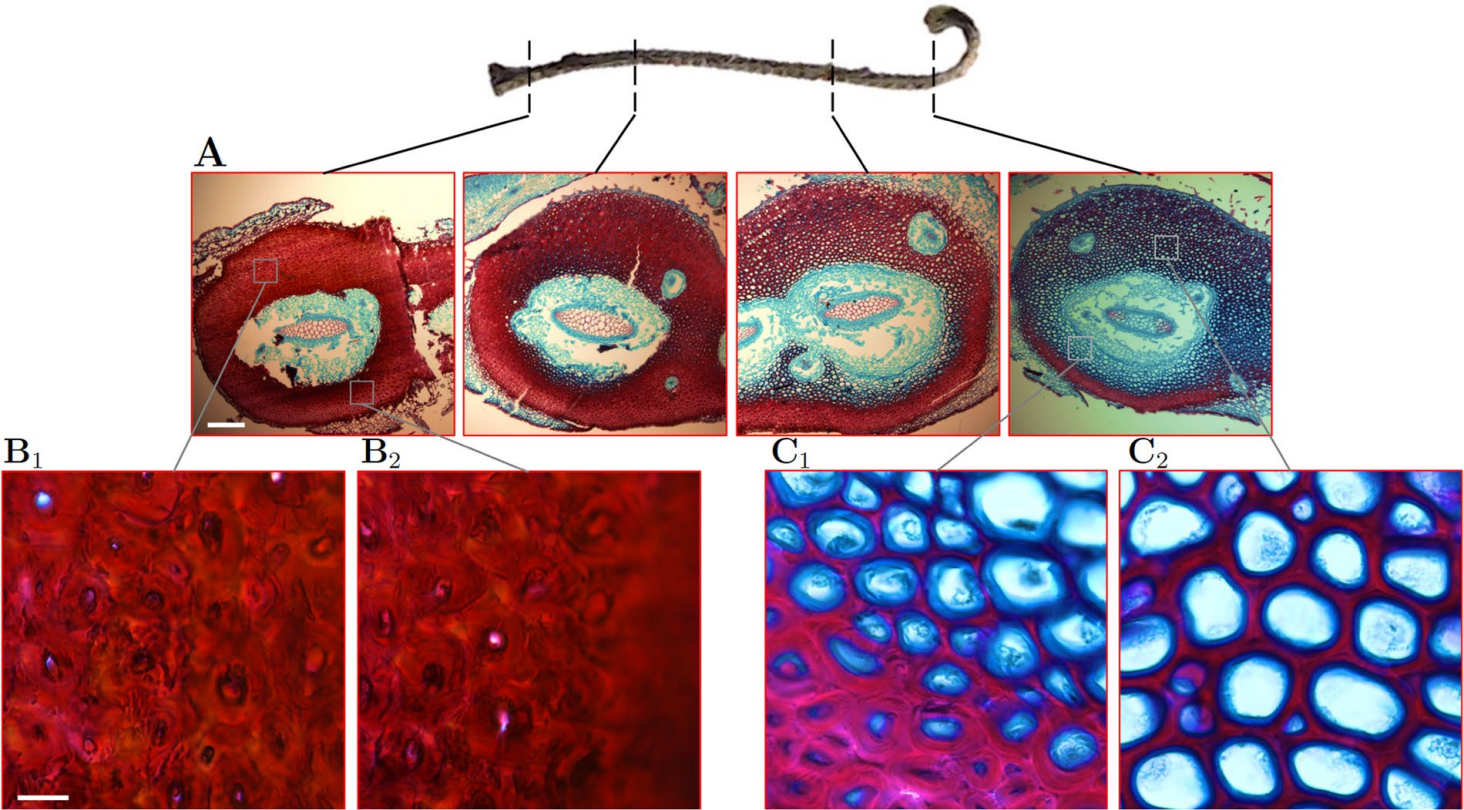
*S*. *lepidophylla* Cortical Cell Wall Composition. (**A**) Transverse sections from four inner stem regions (apical, apical-middle, middle-basal, and basal) stained with alcian blue (blue), and with safranin O (red) to detect lignin. Stem cortex became increasingly stained for safranin O moving from stem tip to base. At the tip, abaxial cortex is visibly more stained with safranin O than adaxial cortex. At the base, both stem sides appear to stain strongly with safranin O. Apical-middle and middle-basal regions show intermediate staining between what is observed at the stem tip and base. Scale bar: 200 μm. (**B**_**1**_,**B**_**2**_) High magnification images of adaxial and abaxial cell walls in basal stem regions. (**C**_**1**_,**C**_**2**_) High magnification images of abaxial and adaxial cell walls in apical stem regions. In the apical region in adaxial cells, safranin O stains the outer secondary wall layers, while the counterstain, alcian blue, stains the inner secondary wall layer. A similar pattern is seen in abaxial cortical cells near the center of the stem, but cells near the stem periphery show more layers with safranin O staining and a narrow inner layer stained with alcian blue. In contrast, adaxial and abaxial cell wall layers in the basal stem region almost exclusively stain with safranin O, with only a very small inner layer stained with alcian blue. Scale bar: 10 μm.

Cell walls were stained with safranin O to detect polyphenolics, including lignin, and counter-stained with alcian blue to detect other cell wall materials (Fig. 5A,B)^19,56,57^. In all sections, the outermost SCW layers of *S*. *lepidophylla* cortex stained with safranin O, while the innermost SCW layer stained with alcian blue (Fig. 5B). Increasing wall thickness from stem tip to base corresponded with increasing SCW layers stained with safranin O, while alcian blue continued to exclusively stain the innermost SCW layer^11^. Further investigation with other cell wall stains and antibodies revealed that the SCW inner layer stained by alcian blue appears to be predominantly enriched in cellulose (Supplementary Fig. 3) and hemicellulose (Supplementary Fig. 4), but not pectin (Supplementary Fig. 5).

While this compositional pattern does not appear to correspond to the SCW morphological layering observed with AFM and TEM, it does resemble the established bilayer-like cell wall composition of gelatinous (G) fiber and G- fiber-like cells in other species, such as tension wood, coiling vines, and ice plant seed capsules^58–61^. G-fiber and G-fiber-like cells differ from normal fiber cells by the presence of a tertiary hydrophilic, gelatinous layer in their cell wall that is surrounded by a lignified SCW layer. In both tension wood and coiling vines, the gelatinous layer is pectin-rich^58,59^, while in ice plant seed capsules, this layer is predominantly composed of cellulose^60^. In all these species, G-fibers lead to directional organ movement, and, in the case of ice plants, reversible deformation. Given the compositional bilayer similarity, and the (hemi)cellulose inner layer that resembles the cellulose-rich inner layer of ice plant seed capsules cells, *S*. *lepidophylla* cortical cell composition likely contributes to reversible stem deformation through swelling and shrinking of its (hemi)cellulose inner SCW layer. The degree of swelling/shrinking is most likely determined by the proportion of lignin:(hemi)cellulose layers present in the wall, resulting from developmental gradients of SCW lignin deposition and cell wall thickness (i.e., increased SCW lignification and cell wall thickness as cells mature)^38^. A polyphenolic matrix that is deposited around cell wall polysaccharides, lignin could be directly affecting SCW stiffness and/or preventing cell wall hydration/swelling due to its hydrophobicity^11^. In the adaxial apical cortex where walls are thinnest and the proportion of lignin layers is qualitatively similar to that of (hemi)cellulose, swelling probably is not as restricted as it might be in the adaxial basal cortex where cell walls are thicker and the proportion of lignified layers is higher. This would give rise to differential swelling leading to stem curling toward the adaxial side, and a gradient of curling along the stem length.

## Conclusions

*S*. *lepidophylla* is a desiccation tolerant plant used as a model to study hierarchical, water-responsive deformation. Previous studies at the organ and tissue levels suggest morphological and compositional properties leading to reversible stem curling^10,11^. Here, we examine cell wall morphology, mechanics and composition to inform the contribution of cortical SCWs to the overall curling of inner *S*. *lepidophylla* stems. Using AFM, we identified cortical cell wall stiffness gradients from stem tip to base, and also between adaxial and abaxial stem sides (Fig. 3, Table 1). Changes in SCW layer morphology (Fig. 2) and composition (Fig. 5) between adaxial and abaxial stem sides, as well as composition along the stem length, might contribute to the observed elasticity gradients. Further, G-fibre-like bilayer composition of cortical cell walls combined with the presence of perpendicular stiffness gradients could contribute to differential swelling between adaxial and abaxial stem sides and along the stem length, resulting not only in directional bending, but also linear stem curling. These perpendicular gradients likely result from established SCW developmental gradients (e.g., increasing stem tip to base lignification [Fig. 2] and cell wall thickness), and separate adaxial and abaxial differentiation programs^37,38,41^. *S*. *lepidophylla* has provided the opportunity to study three-dimensional gradients at small length scales that lead to macro-level actuation. The cell wall properties identified here could be explored further with computational models to extract features for the development of synthetic actuators with improved performance and structure at the micro-level^2,3^.

## Supplementary information

Supplementary Information.

## References

[1] Burgert I, Fratzl P (2009). Actuation systems in plants as prototypes for bioinspired devices. Philos. Trans. Royal Soc. A.

[2] Naleway SE, Porter MM, McKittrick J, Meyers MA (2015). Structural design elements in biological materials: application to bioinspiration. Adv. Mater..

[3] Lv C (2018). Humidity-responsive actuation of programmable hydrogel microstructures based on 3D printing. Sens. Actuator B-Chem..

[4] Erb RM, Sander JS, Grisch R, Studart A (2013). Self-shaping composites with programmable bioinspired microstructures. Nat. Commun..

[5] Shtein I, Bar-On B, Popper ZA (2018). Plant and algal structure: from cell walls to biomechanical function. Physiol. Plant..

[6] Elbaum R, Abraham Y (2014). Insights into the microstructures of hygroscopic movement in plant seed dispersal. Plant Sci..

[7] Borowska-Wykret D (2017). Gradient of structural traits drives hygroscopic movements of scarious bracts surrounding Helichrysum bracteatum capitulum. Ann. Bot..

[8] Hofhuis H (2016). Morphomechanical innovation drives explosive seed dispersal. Cell.

[9] Brulé V, Rafsanjani A, Pasini D, Western TL (2016). Hierarchies of plant stiffness. Plant Sci..

[10] Rafsanjani A, Brulé V, Western TL, Pasini D (2015). Hydro-responsive curling of the resurrection plant *Selaginella lepidophylla*. Sci. Rep..

[11] Brulé V, Rafsanjani A, Asgari M, Western TL, Pasini D (2019). Three-dimensional functional gradients direct stem curling in the resurrection plant *Selaginella lepidophylla*. J. Royal Soc. Interface.

[12] Lebkuecher JG, Eickmeier WG (1991). Reduced photoinhibition with stem curling in the resurrection plant *Selaginella lepidophylla*. Oecologia.

[13] Lebkuecher JG, Eickmeier WG (1993). Physiological benefits of stem curling for resurrection plants in the field. Ecology.

[14] Niklas, K. J. *Plant biomechanics: an engineering approach to plant form and function* (University of Chicago Press, 1992).

[15] Vincent JFV (1992). Biomechanics–materials.

[16] Cosgrove DJ (2015). Plant cell wall extensibility: connecting plant cell growth with cell wall structure, mechanics, and the action of wall-modifying enzymes. J. Exp. Bot..

[17] Milani P, Braybrook SA, Boudaoud A (2013). Shrinking the hammer: micromechanical approaches to morphogenesis. J. Exp. Bot..

[18] Vogler H, Felekis D, Nelson BJ, Grossniklaus U (2015). Measuring the mechanical properties of plant cell walls. Plants.

[19] Ruzin SE (1999). Plant microtechnique and microscopy.

[20] Young RE (2008). Analysis of the Golgi apparatus in Arabidopsis seed coat cells during polarized secretion of pectin-rich mucilage. Plant Cell.

[21] McCartney L, Marcus SE, Knox JP (2005). Monoclonal antibodies to plant cell wall xylans and arabinoxylans. J. Histochem. Cytochem..

[22] Knox JP, Linstead PJ, King J, Cooper C, Roberts K (1990). Pectin esterification is spatially regulated both within cell walls and between developing tissues of root apices. Planta.

[23] Knox JP, Linstead PJ, King J, Cooper JPC, Roberts K (1991). Developmentally regulated epitopes of cell surface arabinogalactan proteins and their relation to root tissue pattern formation. Plant J..

[24] Gierlinger N, Keplinger T, Harrington M (2012). Imaging of plant cell walls by confocal Raman microscopy. Nat. Protoc..

[25] Hutter JL, Bechhoefer J (1993). Calibration of atomic-force microscope tips. Rev. Sci. Instrum..

[26] Walters D (1996). Short cantilevers for atomic force microscopy. Rev. Sci. Instrum..

[27] Johnson, K. L. *Contact Mechanics* (Cambridge University Press, 1987).

[28] Asgari M (2017). Micro-mechanical, continuum-mechanical, and AFM-based descriptions of elasticity in open cylindrical micellar filaments. Soft Matter.

[29] Asgari M, Abi-Rafeh J, Hendy GN, Pasini D (2019). Material anisotropy and elasticity of cortical and trabecular bone in the adult mouse femur via AFM indentation. J. Mech. Behav. Biomed. Mater..

[30] Latifi N, Asgari M, Vali H, Mongeau L (2018). A tissue-mimetic nano-fibrillar hybrid injectable hydrogel for potential soft tissue engineering applications. Sci. Rep..

[31] Gibson LJ (2012). The hierarchical structure and mechanics of plant materials. J. R. Soc. Interface.

[32] Burgert, I. & Dunlop, J. W. C. Micromechanics of cell walls. *Mechanical integration of plant cells and plants*, Springer, 27–52 (2011).

[33] Zhong R, Ye Z-H (2014). Secondary cell walls: biosynthesis, patterned deposition and transcriptional regulation. Plant Cell Physiol..

[34] Liu D, Song J, Anderson DP, Chang PR, Hua Y (2012). Bamboo fiber and its reinforced composites: structure and properties. Cellulose.

[35] Parameswaran N, Liese W (1976). On the fine structure of bamboo fibres. Wood Sci. Technol..

[36] Zou L, Jin H, Lu W-Y, Li X (2009). Nanoscale structural and mechanical characterization of the cell wall of bamboo fibers. Mater. Sci. Eng. C..

[37] Tan T (2011). Mechanical properties of functionally graded hierarchical bamboo structures. Acta Biomater..

[38] Zhong R, Taylor JJ, Ye Z-H (1997). Disruption of interfascicular fiber differentiation in an Arabidopsis mutant. Plant Cell.

[39] Habibi MK, Samaei AT, Gheshlaghi B, Lu J, Lu Y (2015). Asymmetric flexural behavior from bamboo’s functionally graded hierarchical structure: underlying mechanisms. Acta Biomater..

[40] Persson, K. Micromechanical modelling of wood and fibre properties. *Dissertation*, *Lund University* (2000).

[41] Barra-Jiménez A, Ragni L (2017). Secondary development in the stem: when Arabidopsis and trees are closer than it seems. Curr. Opin. Plant Biol..

[42] Dixon PG, Gibson LJ (2014). The structure and mechanics of Moso bamboo material. J. R. Soc. Interface.

[43] Gardiner B, Berry P, Moulia B (2016). Wind impacts on plant growth, mechanics and damage. Plant Sci..

[44] Ghavami K, Rodrigues CS, Paciornik S (2003). Bamboo: functionally graded composite material. Asian J. Civil Eng..

[45] Fukuda H (2004). Signals that control plant vascular cell differentiation. Nat. Rev. Mol. Cell Biol..

[46] Routier-Kierzkowska A-L (2012). Cellular force microscopy for *in vivo* measurements of plant tissue mechanics. Plant Physiol..

[47] Braybrook SA (2015). Measuring the elasticity of plant cells with atomic force microscopy. Methods Cell Biol..

[48] Beauzamy L, Derr J, Boudaoud A (2015). Quantifying hydrostatic pressure in plant cells by using indentation with an atomic force microscope. Biophys. J..

[49] Malgat R, Faure F, Boudaoud A (2016). A mechanical model to interpret cell-scale indentation experiments on plant tissues in terms of cell wall elasticity and turgor pressure. Front. Plant Sci..

[50] Asgari M, Latifi N, Heris HK, Vali H, Mongeau L (2017). *In vitro* fibrillogenesis of tropocollagen type III in collagen type I affects its relative fibrillar topology and mechanics. Sci. Rep..

[51] Engelund ET, Svensson S (2011). Modelling time-dependent mechanical behaviour of softwood using deformation kinetics. Holzforschung.

[52] Yamashita M, Yoshida M, Matsuo M, Sato S, Yamamoto H (2016). Observations of Wood Cell Walls with a Scanning Probe Microscope. Mater. Sci. Appl..

[53] Keryvin V (2015). Analysis of flax fibres viscoelastic behaviour at micro and nano scales. Compos Part A Appl. Sci. Manuf..

[54] Cosgrove DJ, Jarvis MC (2012). Comparative structure and biomechanics of plant primary and secondary cell walls. Front Plant Sci..

[55] Bidhendi AJ, Geitmann A (2015). Relating the mechanics of the primary plant cell wall to morphogenesis. J. Exp. Bot..

[56] Bond J, Donaldson L, Hill S, Hitchcock K (2008). Safranine fluorescent staining of wood cell walls. Biotech. Histochem..

[57] Marjamaa K (2003). Developmental lignification and seasonal variation in *β*-glucosidase and peroxidase activities in xylem of Scots pine, Norway spruce and silver birch. Tree Physiol..

[58] Bowling AJ, Vaughn KC (2008). Immunocytochemical characterization of tension wood: gelatinous fibers contain more than just cellulose. Am. J. Bot..

[59] Bowling AJ, Vaughn KC (2009). Gelatinous fibers are widespread in coiling tendrils and twining vines. Am. J. Bot..

[60] Harrington MJ (2011). Origami-like unfolding of hydro-actuated ice plant seed capsules. Nat. Commun..

[61] Meloche CG, Knox JP, Vaughn KC (2007). A cortical band of gelatinous fibers causes the coiling of redvine tendrils: a model based upon cytochemical and immunocytochemical studies. Planta.

