# Supplementary material for: Nano-indentation reveals a potential role for gradients of cell wall stiffness in directional movement of the resurrection plant Selaginella lepidophylla
Source: Sci Rep. 2020 Jan 16;10:506. doi: 10.1038/s41598-019-57365-z (PMC6965169; doi:10.1038/s41598-019-57365-z)
Supplement: Supplementary file 1 — Supplementary Information. [file 41598_2019_57365_MOESM1_ESM.pdf]

SUPPLEMENTARY MATERIAL FOR: Nano-indentation reveals a potential role for gradients of cell wall stiffness in directional movement of the resurrection plant *Selaginella lepidophylla*

Meisam Asgari*,[1,2] Véronique Brulé†,[3] Tamara L. Western,[3] and Damiano Pasini‡[1]

[1]Department of Mechanical Engineering, McGill University, 817 Sherbrooke Street West, Montréal, QC H3A 0C3, Canada

[2]Theoretical and Applied Mechanics Program, School of Engineering and Applied Science, Northwestern University, 2145 Sheridan Rd., Evanston, IL 60208-3109, USA

[3]Department of Biology, McGill University, 1205 Avenue Docteur Penfield, Montréal, QC H3A 1B1, Canada

[1] Mitra, P. P. & Loqué, D. Histochemical staining of Arabidopsis thaliana secondary cell wall elements. *J. Vis. Exp.* **87**, e51381 (2014).

[2] Pattathil, S. *et al.* A comprehensive toolkit of plant cell wall glycan-directed monoclonal antibodies. *Plant Phys.* **153(2)**, 514–525 (2010).

[3] Hall, H. C., Cheung, J. & Ellis, B. E. Immunoprofiling reveals unique cell-specific patterns of wall epitopes in the expanding Arabidopsis stem. *Plant J.* **74**, 134–147 (2013).

* Co-first author

† Co-first author

‡ Corresponding author, Email: damiano.pasini@mcgill.ca

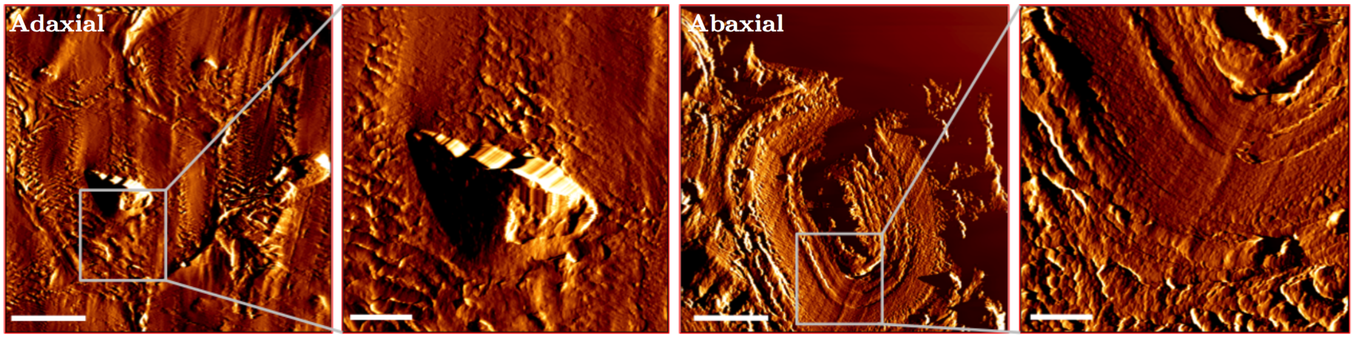

FIG. 1: **Topological Scans of Adaxial and Abaxial Cortical Cells/Cell Walls in the Middle Region of Inner *S. lepidophylla* Stems.** Similar to the rest of the stem, cells in the middle region are round to oval in shape. Cell walls resemble those of the basal region, and abaxial walls show more distinct layering than adaxial cell walls. Scale bars: 7.5 µm for low magnification, and 2.5 µm for high magnification.

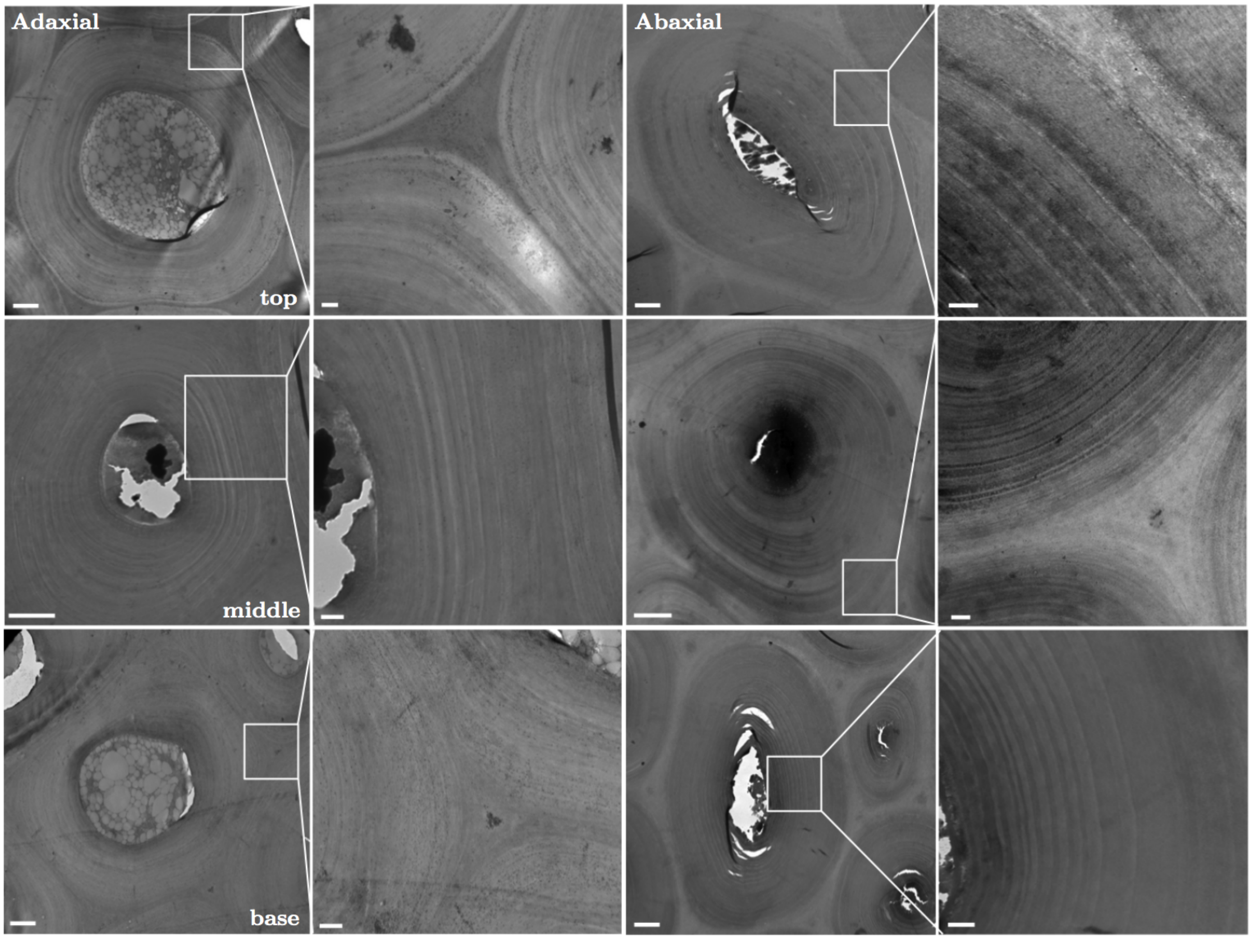

FIG. 2: **Cell Wall Layering Visualized by Transmission Electron Microscopy.** Adaxial (left) and abaxial (right) cortical cells from top, middle and basal stem regions appear round to oval in shape. Higher magnification images reveal cell wall layering. Layering is more prominent in abaxial cortical cell walls along the length of the stem as compared to adaxial cortical cell walls. Scale bars: 2 µm for low magnification, 500 nm for high magnification.

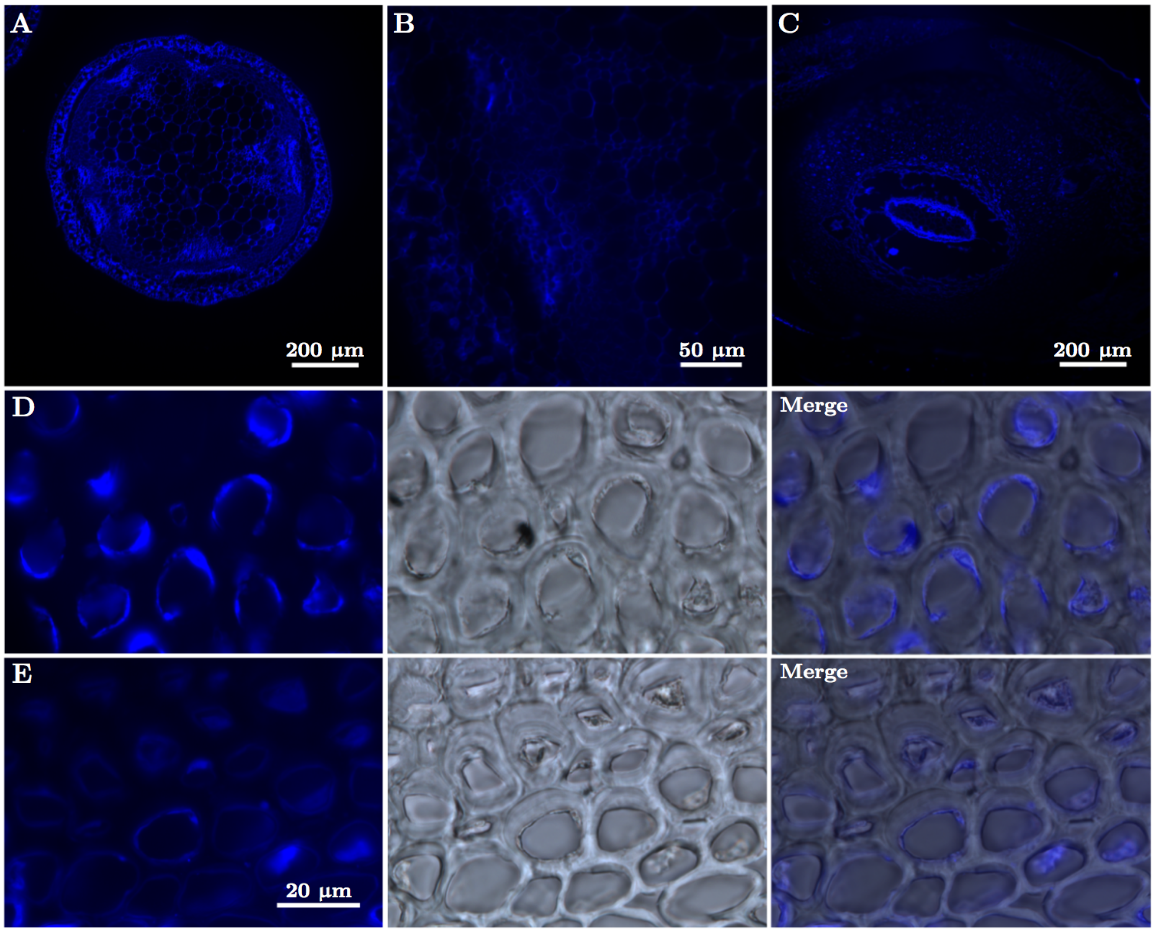

FIG. 3: **Cellulose Cell Wall Distribution.** (A–B) *A. thaliana* (Landsberg) apical stem cross-section as a control for Calcofluor White (CFW) staining [1]. Beta-glycans, primarily cellulose, are indicated by a blue-white colour. CFW binds to most tissue types in *Arabidopsis* stems, and binds most strongly to the phloem cap and xylem. (C–E) *S. lepidophylla* apical stem cross-section. (C) CFW binds to both (D) adaxial and (E) abaxial cortex, as well as to the phloem. CFW binds strongly to the innermost secondary cortical cell wall layer (observed in merged images).

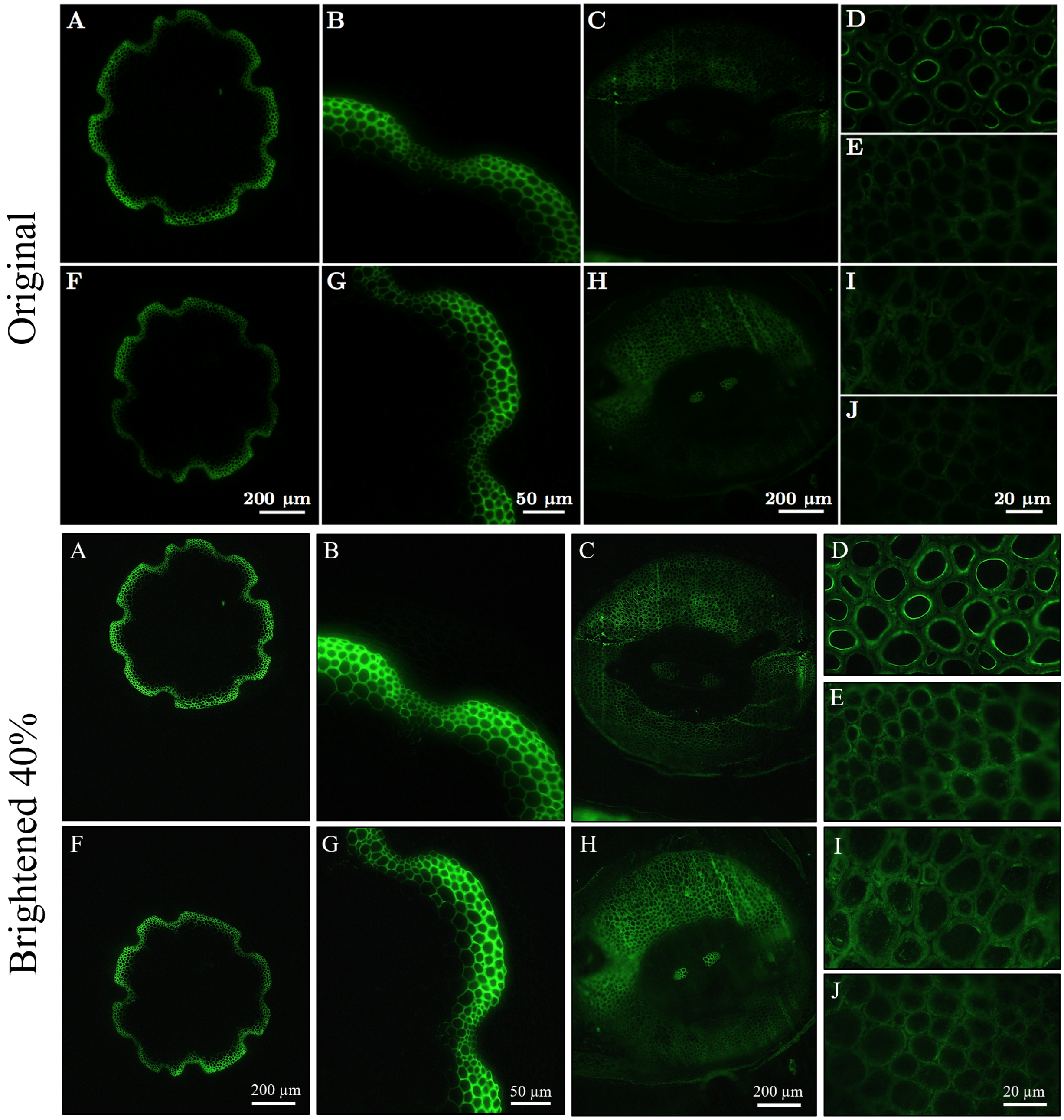

FIG. 4: **Hemicellulose Cell Wall Distribution.** To improve the visualization of the image details, a second set of images with adjusted brightness have been included in addition to the original image set. (A–E) LM10 binding pattern. (A–B) Arabidopsis (Columbia-0) apical stem cross-section as a control for LM10 binding pattern [2]. (C–E) *S. lepidophylla* apical stem cross-section. (C) LM10 binds to cortical tissue and xylem. (D) Adaxial and (E) abaxial cortex. LM10 binds most strongly to the innermost secondary cell wall layer in adaxial cortex, and binds throughout the rest of the secondary cell wall in both adaxial and abaxial cortex to a lesser degree. (F–J) LM11 binding pattern. (F–G) Arabidopsis (Columbia-0) apical stem cross-section as a control for LM11 binding pattern [2]. (C–E) *S. lepidophylla* apical stem cross-section. (C) LM11 binds to cortical tissue and xylem. (D) Adaxial and (E) abaxial cortex. LM11 binds throughout the secondary cell wall in both adaxial and abaxial cortex.

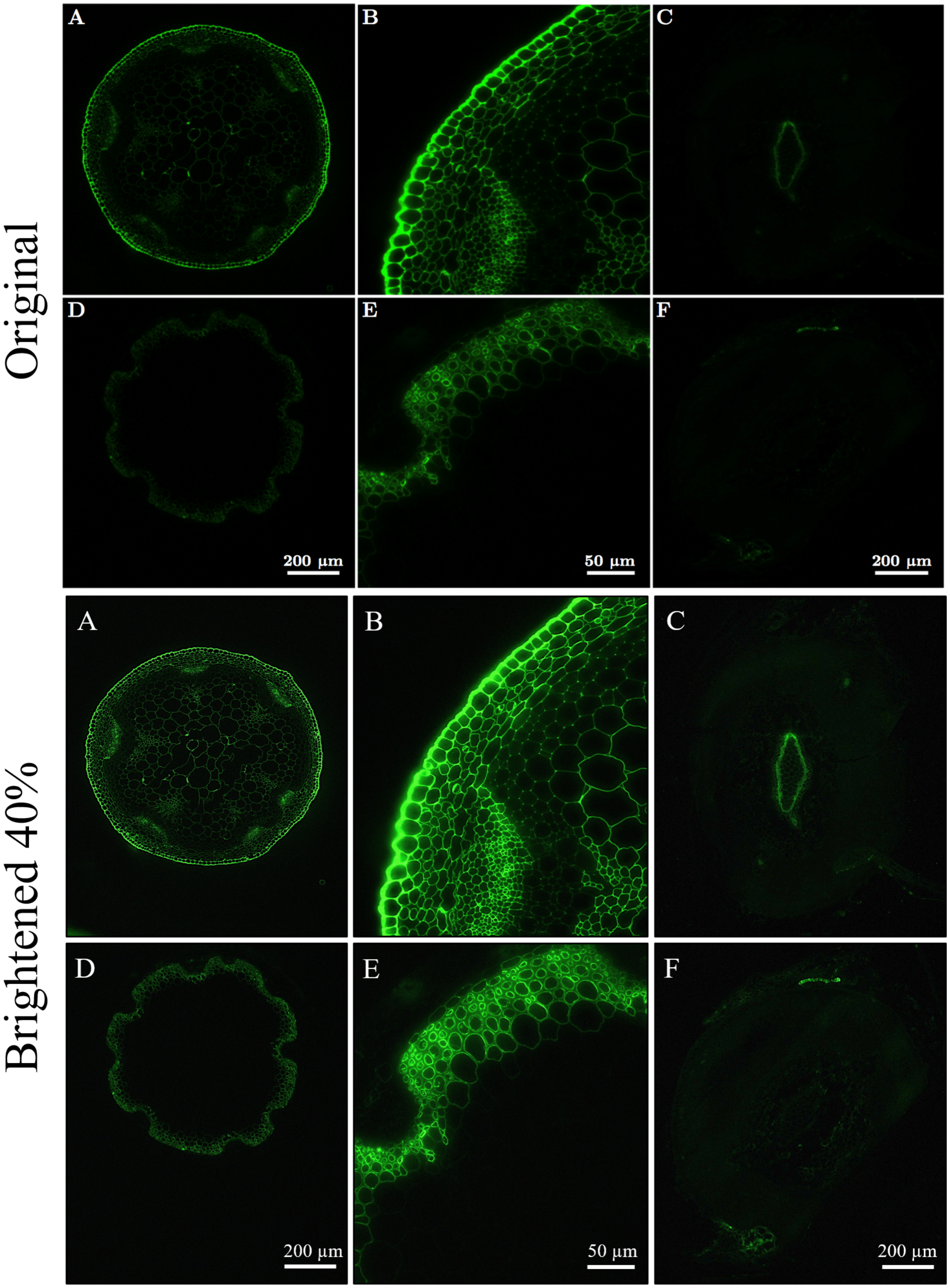

FIG. 5: **Pectin Cell Wall Distribution.** To improve the visualization of the image details, a second set of images with adjusted brightness have been included in addition to the original image set. (A–C) JIM7 binding pattern. (A–B) Arabidopsis (Columbia-0) apical stem cross-section as a control for JIM7 binding pattern [3]. (C) *S. lepidophylla* apical stem cross-section. JIM7 binds to phloem primary cell walls and the middle lamella of the xylem. (D–F) JIM13 Binding pattern. (D–E) Arabidopsis (Columbia-0) apical stem cross-section as a control for JIM13 binding pattern [3]. (F) *S. lepidophylla* apical stem cross-section. JIM13 binds in some places to the epidermis. Neither JIM7 nor JIM13 bind to cortex cell walls or the middle lamella between cortical cells.
